# Harnessing the Power of Behavioral Science: An Implementation Pilot to Improve the Quality of Maternity Care in Rural Madagascar

**DOI:** 10.9745/GHSP-D-23-00007

**Published:** 2023-11-30

**Authors:** Jana Smith, Marie Sandra Lennon, Madeline Kau, Anja Noeliarivelo Ranjalahy, Liliane Ingabire, Charlotte Warren, Sara V. Flanagan

**Affiliations:** a ideas42, New York, NY, USA.; b ideas42, Antananarivo, Madagascar.; c TANDEM S.A.R.L., Antananarivo, Madagascar.; d Accessible Continuum of Care and Essential Services Sustained, Antananarivo, Madagascar.; e Population Council, New York, NY, USA.

## Abstract

**Background:**

Postpartum hemorrhage (PPH) is the leading direct cause of maternal deaths worldwide, and women in low-income countries are at particularly high risk of dying from PPH-related consequences. Most deaths can be avoided through consistent provider adherence to prevention protocols and timely, appropriate management, yet providers do not consistently adhere to these best practices.

**Using Behavioral Design To Develop Solutions to Improve Provider Care:**

We applied the behavioral design methodology to identify behavioral drivers, develop solutions, and build a program theory of change. Implementation research was conducted to understand the adoption, desirability, feasibility, and appropriateness of the solutions and explore suggestive findings related to impact. Data were collected through observation and in-depth interviews. Solutions developed included: (1) a timer to remind providers of the 1-minute window to administer oxytocin; (2) a glow-in-the-dark poster illustrating a simplified algorithm for PPH management; (3) badges to assign family members tasks to support providers during labor and delivery; and (4) a risk visualization exercise. Clinical mentors introduced the solutions during facility visits, and providers received orientation using videos. Solutions were piloted in 10 rural facilities in southeastern Madagascar during November–December 2020.

**Results:**

Providers reported high adoption of the timers and task badges during routine deliveries. They remarked on the desirability and appropriateness of the timer, task badges, and algorithm poster, as well as the value of the cocreation process. Adoption of the timer solution shows promise in having a potential positive impact on increasing the awareness of and adherence to timely oxytocin administration.

**Conclusion:**

This work highlights the promise of applying behavioral science to identify underlying drivers of gaps in clinical practice and to develop innovative and desirable solutions to address them.

## Abstract (precis)

Applying a behavioral design methodology resulted in cocreating 4 innovative solutions to improve provider's compliance with postpartum hemorrhage management protocols in rural Madagascar to help improve the quality of maternity care.

## Teaser

**Key Findings:**

- Using applied behavioral science and a codesign process with health care providers, postpartum women, facility in-charges, and community members resulted in cocreation and testing of 4 interventions that aimed to address providers' behavioral barriers to their compliance with management of postpartum hemorrhage (PPH) protocols and improve the quality of care during labor and delivery.
- Providers' adoption of the timers to remind them when to administer oxytocin and of the task badges to allow them to engage family members in supporting them during routine delivery was high.
- Providers reported high levels of satisfaction, appropriateness for the context, and feasibility of use of the timer, task badges, and glow-in-the-dark algorithm poster. However, the risk visualization exercise that intended to convey the importance of adhering to PPH management protocols faced challenges in implementation and delivery caused by the COVID-19 pandemic.

**Key Implications:**

- Policymakers and program designers seeking to improve quality of care should systematically consider challenges from a behavioral lens or employ behavioral evidence to design approaches.
- Behavioral science should be used to identify underlying drivers of gaps in clinical practice and to develop innovative and desirable solutions to address those gaps using a process of codesign.

## BACKGROUND

Postpartum hemorrhage (PPH) is the leading direct cause of maternal deaths worldwide. Women in low-income countries have an increased likelihood of dying from PPH-related consequences.^1^ For example, in Madagascar, a national assessment of 303 health facilities found that direct causes contributed to 84% of maternal deaths reviewed, with PPH being the most common direct cause.^2^ Most deaths can be avoided through consistent provider adherence to prevention protocols and timely, appropriate management. According to the World Health Organization, an effective uterotonic should be used within 1 minute after birth for all births to prevent PPH and is the most important intervention to prevent PPH.^3^

Despite the existence of these guidelines, studies around the world have shown that health providers do not consistently adhere to clinical best practices for PPH care.^4^^–^^7^ In Madagascar, a 2011 survey by the Maternal and Child Health Integrated Program found that although oxytocin was given in 85% of deliveries observed, it was only given within 1 minute of birth in the correct dose and route in 21% of cases.^8^ This same study found that uterotonics were only administered for treatment in half of observed PPH cases, despite uterotonic availability in the facilities. Compliance with all elements for PPH prevention occurred in only 13% of observed deliveries.^8^

Historically, many programming efforts have been focused on ensuring the availability of quality uterotonics and training providers on clinical protocols. Given persistent gaps in compliance with PPH prevention measures, more recent research has sought to understand the underlying drivers of lack of compliance with best practices in PPH prevention, early detection, and timely management.^9^^–^^11^ In Madagascar, qualitative research through in-depth interviews and observation was conducted to identify the specific features in the physical and social environment of providers in rural facilities that may explain practice gaps.^11^ The following findings of that research were used to develop the program we describe in this article.

- Providers' perceived low risk of PPH may influence their compliance with best practices, subconsciously or explicitly, and lead them to undervalue the importance of PPH prevention and monitoring measures.
- Providers lack clear feedback on specific components of their performance, which ultimately inhibits continuous improvement of compliance with best practices.
- Providers demonstrate great resourcefulness while operating in a challenging context with limited equipment, supplies, and support; however, overcoming these challenges remains their foremost concern, is cognitively taxing, and may ultimately affect clinical decision-making.

Qualitative research conducted in Madagascar revealed that providers' perceived low risk of PPH may influence their compliance with best practices.

These behavioral insights highlight opportunities to develop and test new approaches to improve provider compliance with PPH prevention and management protocols that go beyond provider training and supportive supervision by exploring behavioral solutions to shift clinical practice. Behavioral science—which includes psychology, cognitive science, neurology, behavioral economics, and other disciplines using scientific methods—has been extensively and successfully used to shift provider behavior in many settings.^12^^–^^14^ Although its application to quality-of-care challenges in low-income countries is more nascent, it holds great promise to illuminate often neglected pathways to behavior change and, in turn, generate new evidence-based solutions.

## USING BEHAVIORAL DESIGN TO DEVELOP SOLUTIONS TO IMPROVE PROVIDER CARE

### Behavioral Design Methodology

**FIGURE 1:**
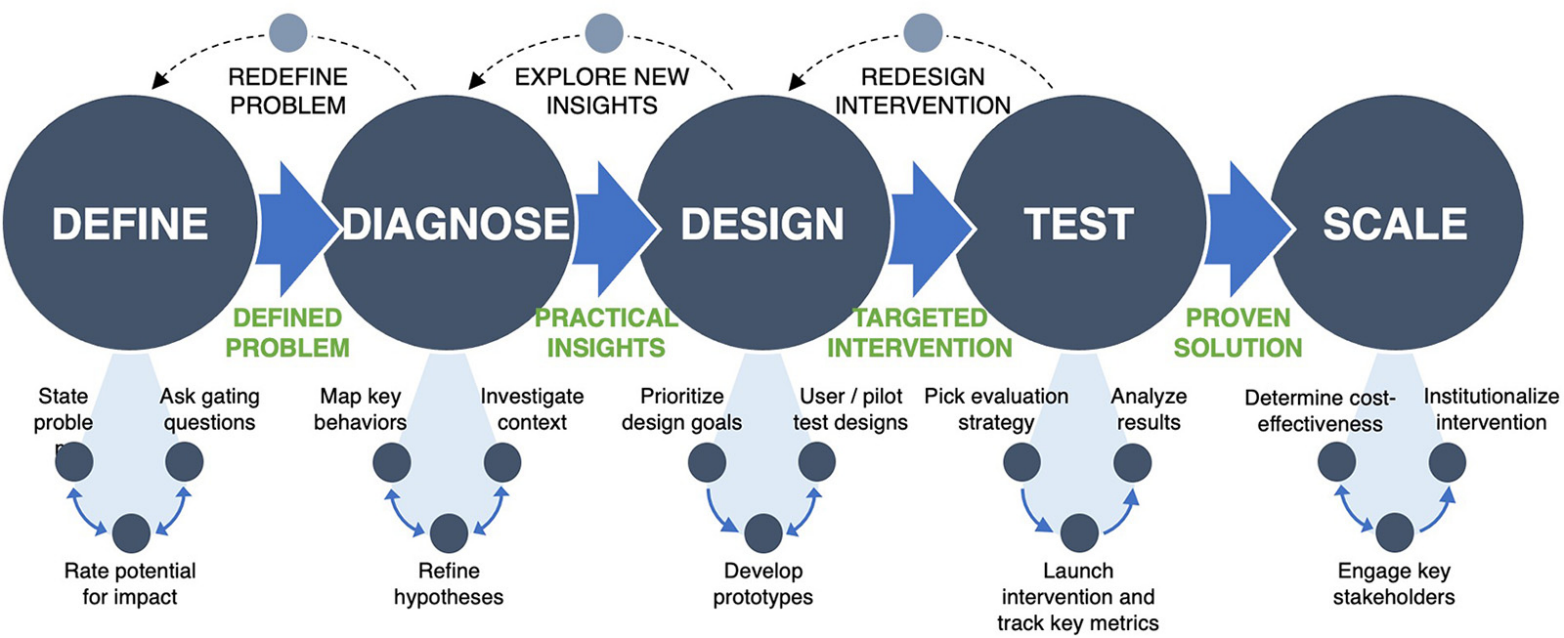
Behavioral Design Methodology Used to Design and Test Solutions to Improve Provider Compliance to Postpartum Hemorrhage Management Protocols

The behavioral design methodology (Figure 1) leverages insights from behavioral economics, social psychology, human-centered design, and other disciplines to develop and test innovative solutions that reshape people's environment to positively influence their behavior.^15^ We applied this methodology to define the behavioral problem, diagnose the behavioral drivers, design solutions, and test these solutions using a theory of change focused on behavioral science. The methods and results from the behavioral diagnosis stage are published elsewhere and are not the focus of this article.^11^

### Design Phase

These findings were used in the design stage to devise question prompts to elicit different solution ideas that could be contextually relevant and linked to the behavioral drivers identified so that the resulting theory of change would be grounded in evidence.

Rough versions of promising interventions were mocked up and further refined and prioritized, on the basis of feasibility and potential for impact, in a cocreation or codesign workshop with government and civil society stakeholders in Madagascar. Project partners indicated what interventions could be implemented within ongoing programming and cost constraints to facilitate scale-up.

The resulting 7 interventions were taken to clinics and communities in the same area where the formative research occurred and—through simulated use and in-depth-interviews—were iteratively refined, discarded, or replaced during user testing in a collaborative codesign process that was conducted with postpartum women, health care workers, facility in-charges, and other community members.

Each of the 4 final chosen solutions sought to address behavioral barriers identified during the diagnosis stage.^11^^,^^17^

**FIGURE 2:**
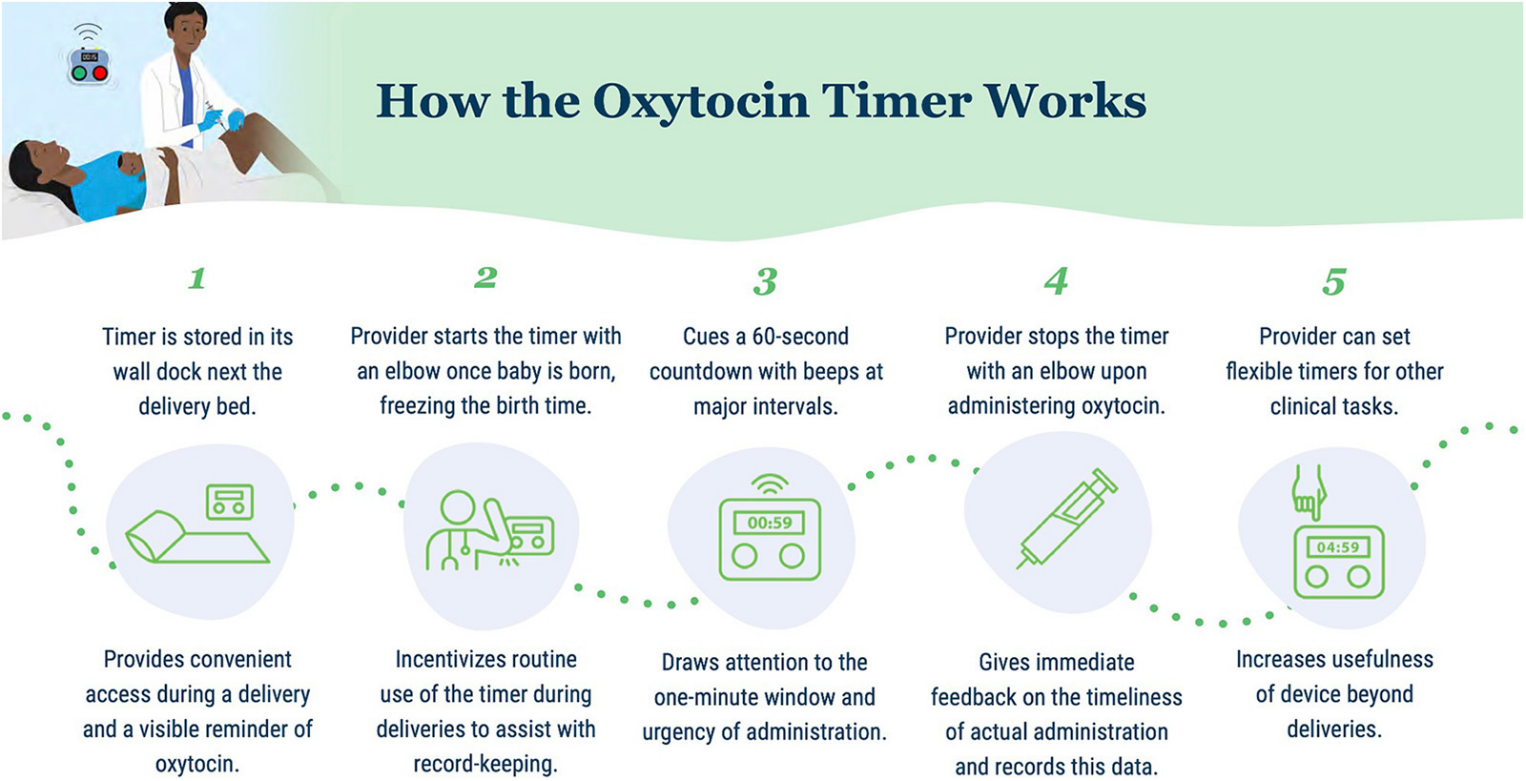
How the Oxytocin Timer Works to Increase Prominence of 1-Minute Window After Birth

**FIGURE 3:**
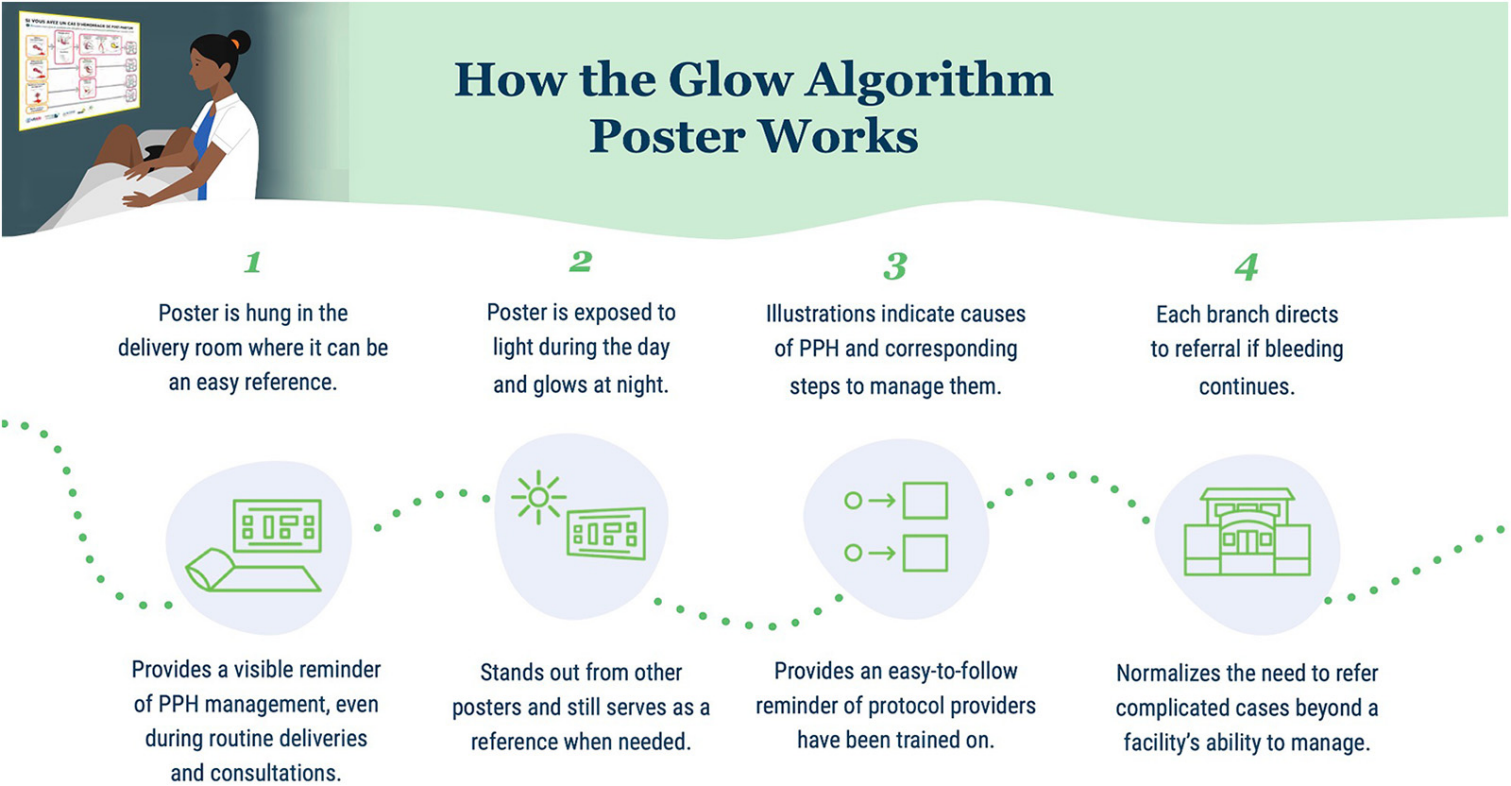
How the Glow-in-the-Dark Algorithm Poster Reminds Providers of PPH Management Protocol Abbreviation: PPH, postpartum hemorrhage.

**FIGURE 4:**
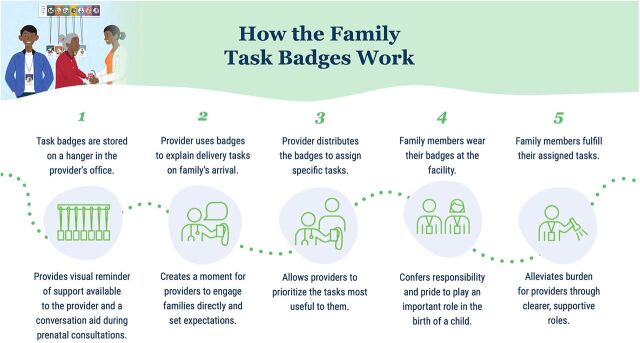
How the Family Task Badges Work to Engage Family to Support Providers and Mothers

**FIGURE 5:**
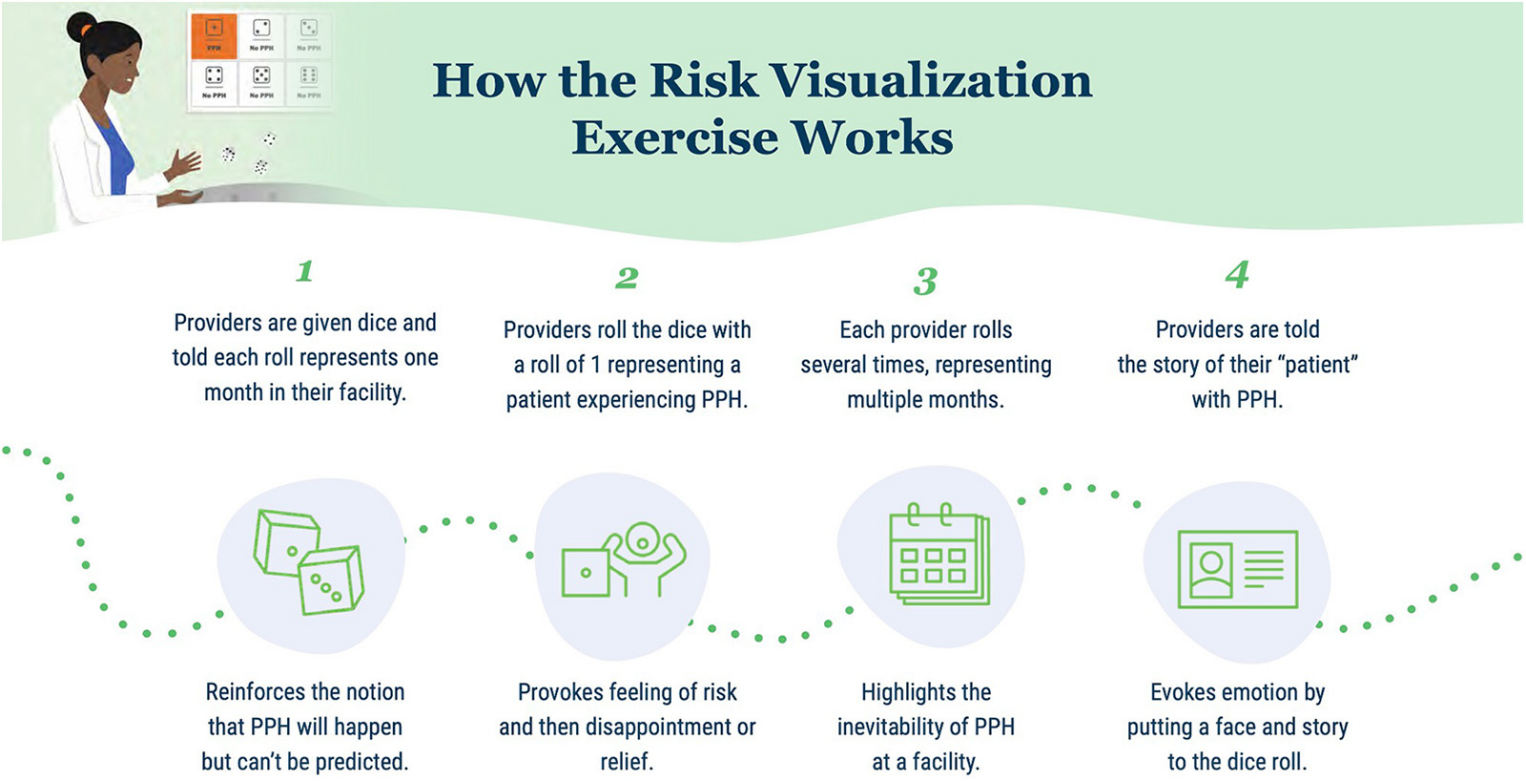
How the Risk Visualization Exercise Motivates Providers to Prevent and Detect PPH Abbreviation: PPH, postpartum hemorrhage.

1. A custom timer sought to cue providers to the 1-minute window to administer oxytocin and provide timely feedback on their adherence to clinical protocol (Figure 2).
2. A glow-in-the-dark algorithm poster with simple and intuitive illustrations to remind providers on PPH management was intended to replace other algorithm posters that providers deemed to be confusing and not easy to reference during labor that providers could see at night in facilities without consistent electricity (Figure 3).
3. A set of family task badges were intended to allow providers with limited support to leverage willing and available family members for specific ways to support providers and mothers by assigning them tasks including providing emotional support, food and water, runner, bleeding monitor, baby care, torch bearer, and cleaner (Figure 4).
4. A risk visualization exercise was intended to heighten the perceived risk of PPH during delivery, the consequences of a client experiencing PPH, and therein the importance of compliance with PPH prevention and management protocol and frequent monitoring for providers (Figure 5).

Each of the final chosen solutions sought to address behavioral barriers identified during the diagnosis stage.

The user testing of these solutions in clinics with providers and community members was particularly important to ensuring the interventions fit the context. With their feedback, several potential solutions beyond the final 4 were dropped, the tasks on the badges evolved significantly, and new features were identified for the timer in addition to modifications to its color and shape.

**Figure:**
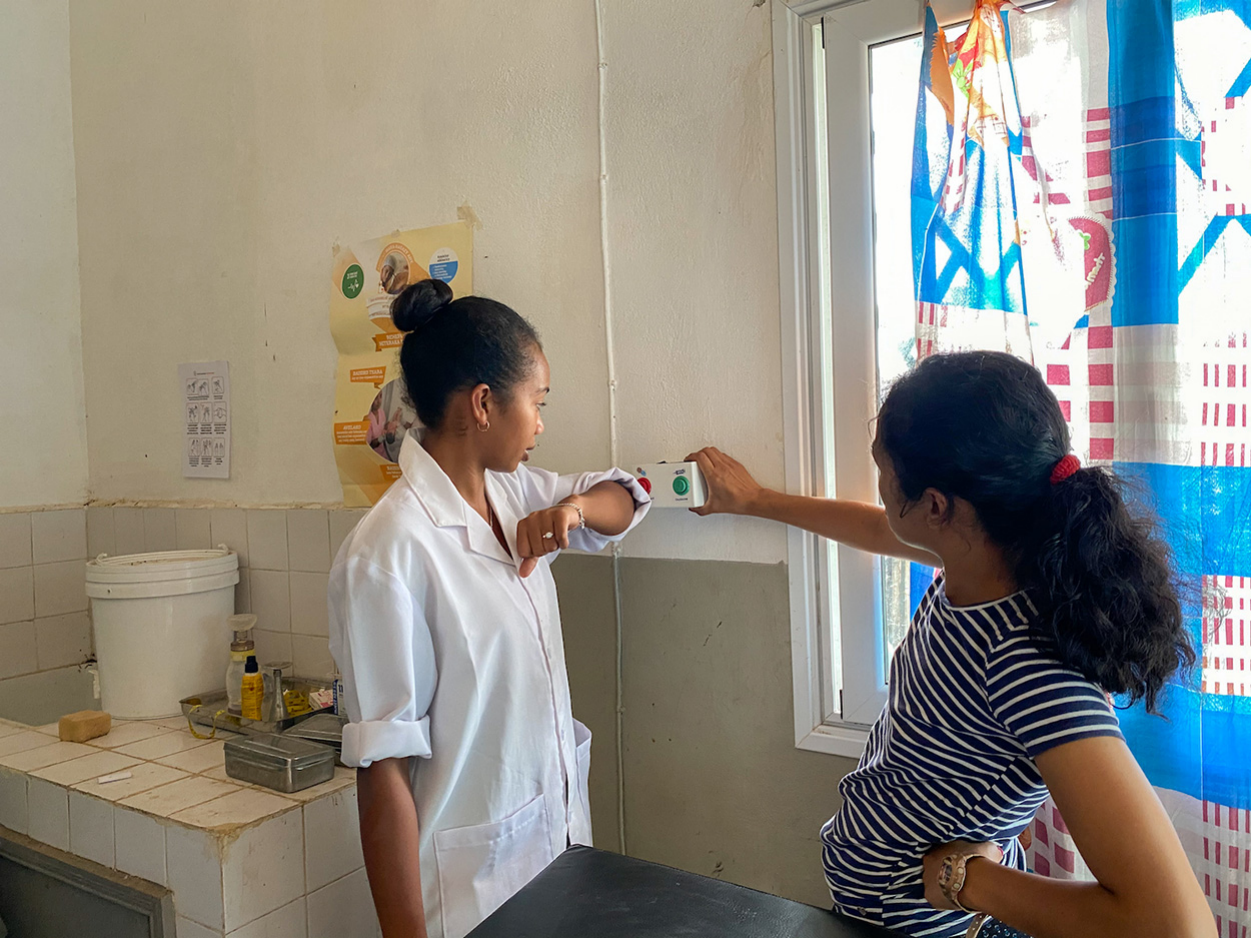
A research team member engages with a facility-based provider in a codesign activity of the oxytocin timer. © 2020 Madeline Kau/ideas42

The solutions that were dropped included an emergency help hotline for clinical guidance, a peer comparison performance feedback poster on oxytocin administration, and a commitment statement between the district health office and facilities with an action plan. The first idea was deemed very desirable by health care workers but was not deemed feasible to implement within the tight project timeline by the partner. The performance feedback poster was discarded because, during user testing, it became clear that there was no reliable source of this data, and there would be significant hassles in preparing this poster each month, such that it was unlikely to be implemented with fidelity. The commitment device was dismissed because district health officials did not perceive that they had decision-making power over the requests that facilities were making, and facility staff generally felt uncomfortable about making demands of the district health office given existing power dynamics that would have required additional implementation strategies outside of the possibilities of the project. The codesign process with different stakeholders was critical to early dismissal of ideas that were not feasible and unlikely to have intended results.

**FIGURE 6:**
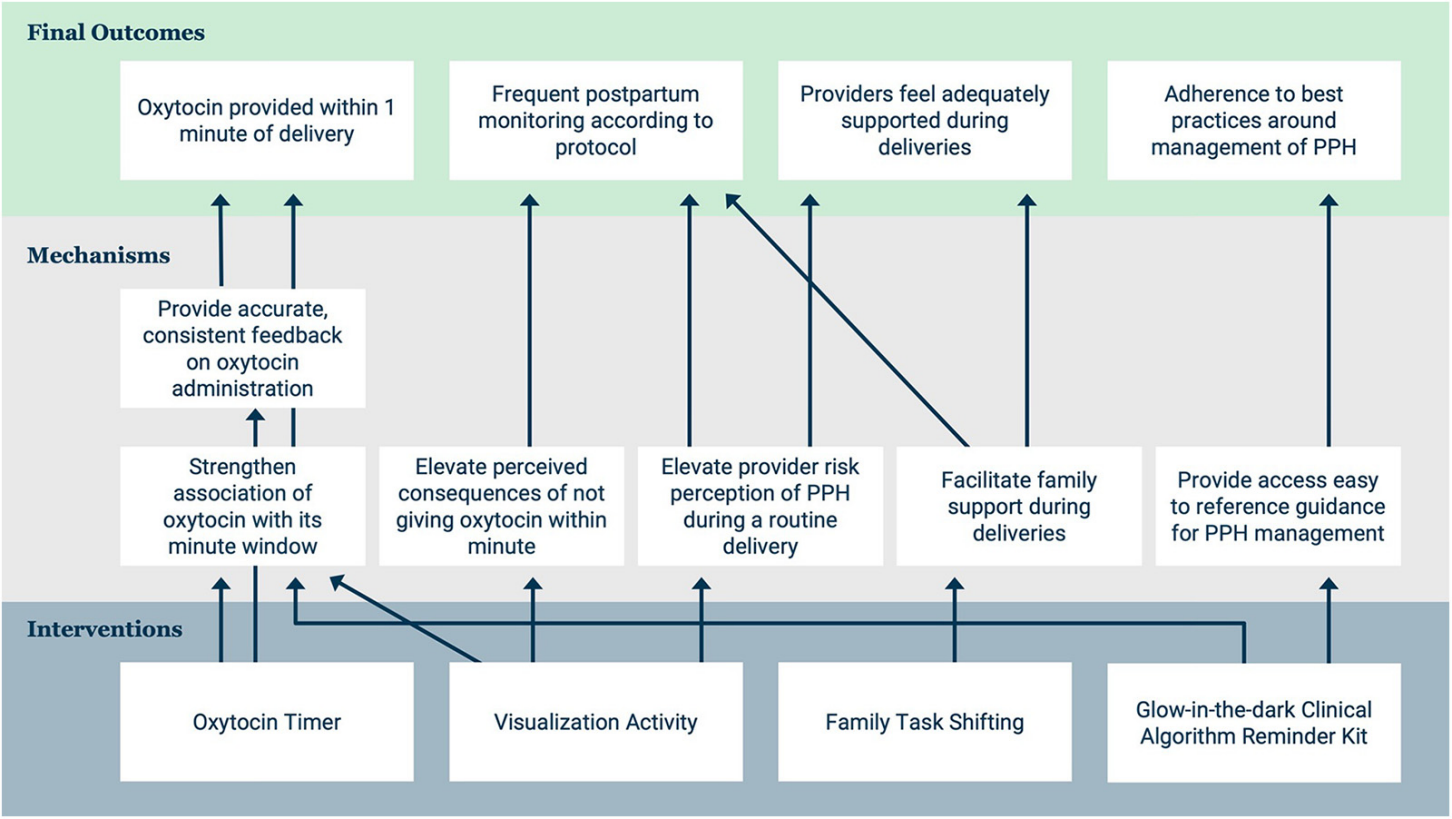
Theory of Change on Package of Solutions to Improve PPH Care Abbreviation: PPH, postpartum hemorrhage.

The resulting theory of change of this package of solutions is shown in Figure 6. It should be noted that diagnosis findings were used as the foundation for articulating how the solutions could have an impact on PPH care. Final outcomes were selected based on hypothesized relevance to improved quality of PPH care and assumptions of what could be reasonably shifted within the short timeline of the project.

The implementation of solutions occurred in 10 rural facilities from 2 districts in southeastern Madagascar through clinical mentors managed by the Accessible Continuum of Care and Essential Services Sustained project funded by the U.S. Agency for International Development. First, clinical mentors received a virtual orientation on each of the solutions and how they worked using prerecorded videos in Malagasy. During visits to participating facilities as part of the broader mentorship intervention implemented by an Advancements in Postpartum Hemorrhage Care project partner, clinical mentors used the same videos to facilitate an adapted version of the risk visualization exercise and to introduce the other 3 solutions to health care workers who regularly attend deliveries. The videos included cues to pause for practicing and role-playing use of the tools and directions to install the materials in the labor ward of the facility. These facilities generally had 1–3 health care workers on staff, were located anywhere from 30 to 90 minutes by car from a more central town (when roads were accessible), and had limited or no access to running water, cell service, or electricity. Mentors did not have additional engagement beyond the initial introduction of the tools.

### Test Phase

To test the interventions, an implementation research study was designed to evaluate the implementation process and the results of a broader package of PPH care solutions that included these interventions and a mentorship intervention designed by another partner on the Advancements in Postpartum Hemorrhage Care project funded by the U.S. Agency for International Development. Specific to interventions described in this article, the implementation research sought to understand the adoption, desirability (acceptability), feasibility, and appropriateness of the solutions.^16^ Given the short time frame and small scale of the research and its design, it was not possible to evaluate impact of the solutions on PPH incidence and management. Instead, research questions were formulated to qualitatively explore components of the theory of change and identify findings suggestive of impact that could be subsequently explored through experimental or quasi-experimental studies.

Our research sought to understand the adoption, desirability (acceptability), feasibility, and appropriateness of a package of PPH care solutions.

Solutions were introduced to providers at 10 rural primary health care facilities in the original study area comprising 2 districts in southeastern Madagascar, and their implementation was monitored over a 2-month pilot period, November–December 2020. Data on provider adoption and experience with the solutions were collected through observation checklists during 3 follow-up visits to the 10 facilities (n=30), all conducted after solution introduction, and 2 rounds of in-depth interviews with the primary provider at each facility (n=10) during the pilot. It should be noted that in most of these facilities, there was only 1 provider in charge of delivery care; this is the person who engaged with the interventions and who was interviewed. An additional round of qualitative interviews with providers and family members of clients (n=8) was conducted in this same area during January–February 2021 as part of an endline survey of PPH knowledge and self-efficacy, relevant for the training and mentorship solutions specifically of the Advancements in Postpartum Hemorrhage Care project, and those transcripts were reviewed for mention of the solutions, as well.

Interviews were conducted in Malagasy and audio-recorded and then transcribed and translated into English. The researchers then coded translated transcripts, and relevant data were excerpted and charted against a thematic framework based on the prioritized implementation outcomes and research questions for final analysis. Each respondent gave their written informed consent for the various data collection activities, and measures were taken to preserve the confidentiality of the data.

### Ethical Approval

The study was approved by the Ethics Committee for Biomedical Research in Madagascar and by the Ethics Committee of the Population Council in the United States.

## RESULTS

Results of the test phase for the oxytocin timer, glow-in-the-dark algorithm poster, family task badges, and risk visualization exercise are organized based on the relevant implementation outcomes (e.g., adoption) or promise of potential impact as per the theory of change.

### Adoption of Interventions

Adoption of the oxytocin timer was high. Data from observation showed that during 78% of the visits, the timer had been used in the previous 24 hours. Implementation facilities were low-volume rural facilities, thus suggesting that the use of the timer was frequent because we did not expect there to be births in all facilities within 24 hours. Health providers also self-reported using the timer at every birth. The primary moment of usage was during delivery, though providers shared that they had also used the device's flexible timer feature to measure time related to the sterilization of tools, rapid diagnostic tests, counting pulse, and counting contractions.

Adoption of the glow-in-the-dark algorithm poster could only be assessed through self-report because it is intended for use as a reference during complications; however, providers reported always seeing it, and 1 provider mentioned having used it during a case of PPH.

*To date, we haven't had a case [of PPH] yet. But anyway, every time we pass by it, we do a sort of retraining every time we pass by. Especially when it's at night, its light is a little bit special, it always catches your eye and you read it… It has become a lesson.* —Provider

During all observation visits, the poster was in the delivery room and could be seen clearly from the delivery bed.

Adoption of the family task badges appeared to be high. In 37% of the observation visits, the badges were actively being used or appeared to have been used very recently. There were no active deliveries during the majority of the observation visits; therefore, we did not expect the badges to be in use during all visits. Most providers reported using the badges at every birth and assigning badges based on their personal priorities and the number of family members in attendance. They also reported that the “runner” badge was the badge used most often and that family members accept the tasks but do not always wear the badges (because of fear of losing them). It should be noted that providers at 1 of the 10 clinics had forgotten how to use the badges, so they had not used them during the initial period of implementation. Several family members cited performing tasks specified by the provider and indicated on the badges during the deliveries they attended.

The risk visualization exercise was included during the orientation of the solutions to all providers as an exercise to be conducted only once, with participation inherent in the implementation process. Therefore, adoption was not a relevant measure to consider for this intervention.

### Desirability of Interventions

Providers reported high levels of satisfaction with the timer.

*There isn't 1 [thing I don't like about the timer], because the existence of the timer prevents problems. It's the absence of it that creates the problems because we'll be in trouble if we don't have the time.* —Provider

Providers discussed how convenient the timer was to use compared to watches or phones and could not find anything they would change about the timer.

*The fact that I don't have to worry about the baby's birth time. That's what I like most about using the timer. No more looking up the time or looking up anything else. Just press and the baby's birth time is recorded.* —Provider

The algorithm poster was also well received by providers. Providers shared that they appreciated the size, the glow element, and the images.

*Every time I walk into the delivery room, I see it. So as soon as I see it, I immediately have in my mind that if there is bleeding, I refer to that. That's what's in my head. I feel light when I see it, every time I see it, because there is no more holding back or wondering what to do if there is a hemorrhage because it is already there.* —Provider

Both providers and family members reported satisfaction with the badges. Providers shared that the badges allowed them to ensure that all tasks were completed.

*The result of badges in childbirth is that all the work is done on time. Everything is done on time.* —Provider

Providers also remarked on the pride that family members felt in being given a badge.

*They (family members) are happy because it matters to them: “This is my job; I get this badge for this job.” They are really happy. They are delighted. “I wear a badge, I work.”* —Provider

Several caregivers expressed appreciation for the trust that providers showed in them by assigning tasks during the delivery, which made them feel very helpful.

The risk visualization exercise faced challenges in implementation, given adaptations made during the COVID-19 pandemic from a facilitated group exercise to a video-guided activity. Providers reported confusion over the link between rolling the dice and PPH (Figure 5). Some perceived that the dice were a tool for use in the facility rather than part of an illustrative reflection exercise to complete once and reported rolling them periodically. One provider shared skepticism about the risk calculations resulting from the exercise and thought the dice were inappropriate for this purpose.

### Appropriateness of Interventions

The timer was deemed by providers as extremely appropriate for the context.

*It makes my work easier and helps me remember. That way I don't forget what I have to do.* —Provider

Another provider shared the value of the collaborative cocreation process used to design the timer.

*We were asked beforehand what it would look like before giving it to us. Its size, the size of the button, its color, if we can find it easily. Then we were shown pictures of models to see which one was suitable. The chosen model was multiplied and made available to us. This means that the tool given to us is suitable. This is what makes it easy to handle. Its size is normal, it is neither big nor small. It is easy to find and easy to use. It can be hung on the wall; it is easy to find because it is nearby. It comes with batteries, which are rechargeable.* —Provider

Providers shared that the content of the poster was appropriate, as it aligned with their clinical training. Providers also shared the value of having a visible reminder.

*It helps not to panic and to know what to use.* —Provider

*You don't forget about the oxytocin preparation at all before attending the birth. This is already a great advantage as a result of the existence of this poster.* —Provider

Providers noted that task badges were appropriate for the context and useful, particularly during the COVID-19 pandemic.

*It also prevents unnecessary crowds and gatherings. Sharing these badges with them seems like a good idea. That way, everyone will have their responsibility and know what they have to do. In the past, this did not exist, everything was done together. Plus, it's frustrating to be surrounded!* —Provider

Providers also shared that family support, as a result of the badges, has made their work easier and less stressful.

Providers shared that family support, as a result of the badges, has made their work easier and less stressful.

*In general, using badges has made my work more organized. Their help makes the work easier and lighter. It makes my job easier because they help me.* —Provider

*Since the existence of the badges, everything has gone smoothly. I didn't get angry anymore.* —Provider

As described previously, the risk visualization exercise was not appropriate as delivered, whether due to confusion over its purpose or the inappropriateness of the dice game representing PPH risk.

### Feasibility of Interventions

Providers remarked that it was easy to use the timer during deliveries. Providers did not remark on any notable challenges with keeping the batteries charged; this was important to assess because the timer was designed to minimize power usage given resource constraints and a desire to make it as hassle-free to use as possible for providers. Several providers remarked on not having had to charge the batteries yet, while others reported charging the batteries 2 or 3 times per month depending on the volume of deliveries.

Almost all facilities were able to find a location to hang the glow-in-the-dark algorithm poster where it was both visible during deliveries and exposed to light during the day to charge it. Providers reported that the poster glowed and stayed visible at night and that the images and writing were clear. There was 1 facility that struggled to get enough sunlight on the poster in the delivery room and moved it during the day to ensure it was charged at night.

Providers generally found the badges feasible to use as intended. Providers did not report problems with storing the badges, and none had been lost or taken from the facility. Providers appreciated the flexibility of being able to choose which badges to distribute based on the circumstances and the number of people in attendance. One provider remarked on the challenge of using the badges when women arrive at the facility late in labor but still found value in the idea of task assignment.

*There are some who are already pushing, and I can't divide the tasks anymore, but I just tell them: you do this, you do that.* —Provider

It was feasible to incorporate the risk visualization exercise into the orientation of the other solutions. However, it was not clear that the revised implementation process of engaging in the exercise individually by video was a feasible way to implement the solution, given challenges in comprehension and perceived appropriateness.

### Promise of Potential Impact of Interventions

Because we were not able to conduct an impact evaluation, we sought to gather qualitative evidence around some of the intermediate outcomes or mechanisms in our theory of change to assess the promise of potential impact of the interventions. The most important of these outcomes was to increase provision of oxytocin within 1 minute of birth by increasing the prominence of this time window in providers' minds, elevating the perceived consequences of delayed administration, and creating awareness of provider performance in the timeliness of administration. Qualitative evidence suggested shifts in all 3 of these mechanisms. Providers frequently mentioned the 1-minute window in interviews about delivery behavior, and some shared that they had previously not known that they were to administer oxytocin in that time window.

*Since we were using the timer, I respect the first minute. But before that, it is true that I don't wait too long, I administer the oxytocin a few minutes after the expulsion.* —Provider

Related to risk, providers shared the connection between PPH and timely oxytocin administration and oxytocin's preventive role. Providers also remarked on how they used the timer to ensure they were administering oxytocin within the recommended time, and several shared that with the timer, they now always respect the minute window.

We also sought to understand the potential impact of the solutions, particularly the family task badges, on provider perception of support during deliveries. During interviews, providers shared that with the badges, family members do help them to ensure that they are able to complete their work.

*Whenever I see them [family members], I think that I am not alone. That there are people who can help me cope with a woman's birth.* —Provider

We also wanted to know if the badges improved communication between providers and family members, as the formative research revealed that a lack of cooperation and understanding between them can cause serious challenges when a complication requires a woman to be urgently transported to a higher-level facility. Providers commented favorably after using the badges to clarify roles.

*I didn't have to keep asking them to do something because everyone knew what they had to do.* —Provider

*My relationship with the family of the patient has improved a lot. Also, nothing disturbs me anymore. I am at peace.* —Provider

*The benefits are the empowerment of the family. Everyone participates in the tasks… the distribution of badges creates solidarity.* —Provider

As mentioned earlier, family members also perceived task assignment as a sign of the provider's trust in them.

The final mechanism we sought to assess was the ease of reference of first-line treatment pathways for PPH and confidence in applying them. Providers explained how the algorithm poster represented an improvement over previous tools and that it served as a quick reference for how they should handle PPH cases.

*It guides in case of PPH, instead of reading manuals. As before, with the protocols [posters], you still have to read a lot. With the poster, you look at the pictures immediately. The pictures are better, you can see everything.* —Provider

Providers also noted feeling confident that they would know what to do in the case of bleeding because the poster made it easier to remember what to do.

*The positive outcome that the poster has brought to my work is that it has made it easier to manage PPH … I don't have to remember because it's there. It makes it easier to manage.* —Provider

## DISCUSSION

Efforts to improve quality of care have not always systematically considered challenges from a behavioral lens or employed behavioral evidence to design approaches. A recent evidence review has suggested that many strategies used to improve health care provider practice in low- and middle-income countries have minimal impact on the practices they seek to influence.^18^ Furthermore, the most common approaches, training and supervision, only have moderate effects together, and training alone has, on average, small effect sizes.^18^ It is time for quality-of-care efforts to include more targeted drivers of provider behavior, rather than primarily addressing knowledge gaps, and to consistently consider programmatic approaches beyond training and supervision. Insights from the behavioral sciences can illuminate how psychology interacts with features of a provider's environment to influence their consistent application of clinical protocols. Our formative research highlighted a series of specific behavioral factors that explain gaps in clinical practice related to PPH and require intervention beyond training and knowledge. Those insights informed the behavioral solutions described here.

It is time for quality-of-care efforts to include more targeted drivers of provider behavior, rather than primarily addressing knowledge gaps.

In addition to developing more precise interventions to enhance providers' environment in a way that is more supportive of their adherence to clinical protocol, behavioral science can also identify innovative pathways to address identified gaps, particularly in the context of resource constraints. For example, while supportive supervision can be an effective intervention to improve quality of care, consistent supervision can be more resource intensive than many low-income health systems can afford or sustain over time. Our work highlights how alternative forms of performance feedback can be created, in this case through the timer, that do not require the expense of extensive supervision and can rely on data collected passively rather than placing more onus on already busy health providers to collect data for performance supervision. While the timer gave cues and immediate performance feedback to providers during deliveries, we designed it to ensure that all usage data would be recorded. While building the functionality to extract and return these data in useful formats was outside the scope of our project, the promising results of the implementation pilot support the potential of pursuing these avenues to enhance provider performance through innovative behavioral applications of this novel data collection system.

Finally, our article highlights the value of codesign in arriving at desirable solutions with high levels of adoption that are feasible to implement. Many tools focused on health care workers go unused because they are not designed collaboratively and in context to ensure their seamless incorporation into existing habits, workstreams, and environments. When new tools or approaches do not solve a felt problem, they must generate perceptible benefits for the user while also entailing minimal hassles to facilitate adoption. We attribute our high adoption rates to careful attention to detail during our collaborative codesign with the health care workers who shaped the solutions to fit their needs. Whether offering a quick and easy way to record birth times or channeling what might have been a bothersome crowd of relatives waiting around the health facility into an enthusiastic support team, providers immediately recognized the value these solutions could bring to their work. Close collaboration and cocreation with the service delivery partner, the Accessible Continuum of Care and Essential Services Sustained project, also helped to ensure that the interventions fit existing or feasible project channels and could be seamlessly integrated into their well-established operations.

One concern that often arises about codesign is the cost and time required, which is often assumed to be outside the realm of possibility for many projects. First, we would note that in-depth development of solutions from the beginning is not needed in projects for which there are existing, evidence-based solutions that could be rapidly adapted and contextualized. In our case, we found scant inquiry into the behavioral dimensions of clinical care during delivery in general and even less related to labor complications. As a result, we did not find solutions that were ready for adaptation and instead believed that a codesign process was the most efficient means to develop solutions to fit the challenging context where our Malagasy midwife collaborators were working. However, we should note that despite pursuing this option, we went from broad ideation of potential solutions to the concretization of viable prototypes ready for production in the course of only 3 months and with a reasonable budget. This process allowed us to (1) save the time and money of investing in the 3 solutions that were dropped during user testing; (2) ensure feasibility and desirability of solutions before implementation, which were confirmed by pilot findings; and (3) identify opportunities to make certain solutions more cost effective (in this case, the flexible timer function that allowed the timer to be used for other clinical cases). Our experience suggests that, in certain cases, a relatively small up-front investment in time and resources for codesign would repay itself by starting implementation on much more solid footing and with stronger buy-in from stakeholders.

### Limitations

While we find our results promising and suggestive of potential for both successful implementation and impact, our research had a few notable limitations. First, the design of the implementation pilot did not allow us to empirically assess the impact of our interventions. This means that we were only able to assess implementation outcomes, such as the feasibility and desirability of the solutions, rather than actual effect on clinical behavior or broader health outcomes. Furthermore, our interventions were only implemented in 10 relatively low-volume facilities, thus limiting our ability to draw out implementation differences between settings. Finally, given the COVID-19 pandemic, training for implementation occurred remotely and with less involvement in the orientation phase than was planned and was paired with delivery of a more intensive training intervention that may have distracted from the introduction of the tools within facilities. Challenges with the risk visualization exercise may have been due to insufficient iteration during the design phase or to gaps in how providers were oriented to these solutions, as the exercise was introduced virtually without prior testing because of required adaptations to the pandemic context. Unfortunately, our monitoring system did not fully capture the context and reception of the initial exercise and orientation to providers on the tools, which in the case of some facilities, may have resulted in their delayed installation or early misunderstandings about their use. A larger scale, rigorous evaluation of these interventions could address many of these limitations and contribute meaningfully to the much-needed evidence base on effective approaches to improve provider practice in low- and middle-income countries.

## CONCLUSION

We believe our work highlights how insights from behavioral science and a collaborative design process can result in interventions that are highly desirable for the people for whom they are intended and hold strong potential for impact, having been closely tailored to contextually specific behavioral evidence. The systematic incorporation of behavioral science evidence and approaches into quality-of-care efforts could further strengthen their impact and adoption.

## Notes

**Peer Reviewed**

**First published online:** July 28, 2023.

## References

[1] Say L, Chou D, Gemmill A, et al. Global causes of maternal death: a WHO systematic analysis. Lancet Glob Health. 2014;2(6):e323–e333. 10.1016/S2214-109X(14)70227-X. 25103301

[2] Vice Primature Charge de Sante Publique; United Nations Population Fund; UNICEF; World Health Organization; Averting Maternal Death and Disability; Multi-Sector Information Service (MSIS). *Evaluation Des Besoins En Matiere de Soins Obstetricaux et Neonatals d’urgence à Madagascar, Rapport Final, Mars 2010*. MSIS; 2010. Accessed July 10, 2023. https://www.yumpu.com/fr/document/view/26912090/rapport-final-eb-sonu-mars-2010-unfpa-madagascar

[3] World Health Organization (WHO). *WHO Recommendations: Uterotonics for the Prevention of Postpartum Haemorrhage*. WHO; 2018. Accessed July 10, 2023. https://apps.who.int/iris/bitstream/handle/10665/277276/9789241550420-eng.pdf 30645062

[4] Braddick L, Tuckey V, Abbas Z, et al. A mixed-methods study of barriers and facilitators to the implementation of postpartum hemorrhage guidelines in Uganda. Int J Gynaecol Obstet. 2016;132(1):89–93. 10.1016/j.ijgo.2015.06.047. 26475077

[5] Oladapo OT, Akinola OI, Fawole AO, et al; Nigerian AMTSL Group. Active management of third stage of labor: evidence versus practice. Acta Obstet Gynecol Scand. 2009;88(11):1252–1260. 10.3109/00016340903280958. 19824866

[6] Mfinanga GS, Kimaro GD, Ngadaya E, et al. Health facility-based Active Management of the Third Stage of Labor: findings from a national survey in Tanzania. Health Res Policy Syst. 2009;7(1):6. 10.1186/1478-4505-7-6. 19371418 PMC2676279

[7] Vivio D, Fullerton JT, Forman R, Mbewe RK, Musumali M, Chewe PM. Integration of the practice of active management of the third stage of labor within training and service implementation programming in Zambia. J Midwifery Womens Health. 2010;55(5):447–454. 10.1016/j.jmwh.2010.02.015. 20732666

[8] Rasolofomanana JR, Rakotovoa JP, Bazant E, Tripathi V. *Quality of Care of the Prevention and Management of Common Maternal and Newborn Complications in Health Facilities in Madagascar*. Jhpiego, Maternal and Child Health Integrated Program; 2011. Accessed July 10, 2023. https://www.mchip.net/sites/default/files/Madagascar%20QoC%20report%20-%20Final.pdf

[9] Bohren MA, Lorencatto F, Coomarasamy A, et al. Formative research to design an implementation strategy for a postpartum hemorrhage initial response treatment bundle (E-MOTIVE): study protocol. Reprod Health. 2021;18(1):149. 10.1186/s12978-021-01162-3. 34261508 PMC8278177

[10] Smith JM, Currie S, Cannon T, Armbruster D, Perri J. Are national policies and programs for prevention and management of postpartum hemorrhage and preeclampsia adequate? A key informant survey in 37 countries. Glob Health Sci Pract. 2014;2(3):275–284. 10.9745/GHSP-D-14-00034. 25276587 PMC4168639

[11] Flanagan SV, Razafinamanana T, Warren C, Smith J. Barriers inhibiting effective detection and management of postpartum hemorrhage during facility-based births in Madagascar: findings from a qualitative study using a behavioral science lens. BMC Pregnancy Childbirth. 2021;21(1):320. 10.1186/s12884-021-03801-w. 33888075 PMC8063356

[12] Meeker D, Linder JA, Fox CR, et al. Effect of behavioral interventions on inappropriate antibiotic prescribing among primary care practices. JAMA. 2016;315(6):562–570. 10.1001/jama.2016.0275. 26864410 PMC6689234

[13] Asch DA, Troxel AB, Stewart WF, et al. Effect of financial incentives to physicians, patients, or both on lipid levels. JAMA. 2015;314(18):1926–1935. 10.1001/jama.2015.14850. 26547464 PMC5509443

[14] Doctor JN, Nguyen A, Lev R, et al. Opioid prescribing decreases after learning of a patient’s fatal overdose. Science. 2018;361(6402):588–590. 10.1126/science.aat4595. 30093595

[15] Datta S, Mullainathan S. Behavioral design: a new approach to development policy. Rev Income Wealth. 2014;60(1):7–35. 10.1111/roiw.12093

[16] Proctor E, Silmere H, Raghavan R, et al. Outcomes for implementation research: conceptual distinctions, measurement challenges, and research agenda. Adm Policy Ment Health. 2011;38(2):65–76. 10.1007/s10488-010-0319-7. 20957426 PMC3068522

[17] Breakthrough RESEARCH. *Tested Solutions for Prevention of Postpartum Hemorrhage*. ideas42; 2021. Accessed September 30, 2022. https://www.ideas42.org/wp-content/uploads/2021/04/i42-Post-Partum-Hemorrhage-Design-Guide.pdf

[18] Rowe AK, Rowe SY, Peters DH, Holloway KA, Chalker J, Ross-Degnan D. Effectiveness of strategies to improve health-care provider practices in low-income and middle-income countries: a systematic review. Lancet Glob Health. 2018;6(11):e1163–e1175. 10.1016/S2214-109X(18)30398-X. 30309799 PMC6185992

